# Cell Volume Changes Regulate Slick (Slo2.1), but Not Slack (Slo2.2) K^+^ Channels

**DOI:** 10.1371/journal.pone.0110833

**Published:** 2014-10-27

**Authors:** Maria A. Tejada, Kathleen Stople, Sofia Hammami Bomholtz, Anne-Kristine Meinild, Asser Nyander Poulsen, Dan A. Klaerke

**Affiliations:** 1 Department of Physiology and Biochemistry (IKVH), Faculty of Health and Medical Sciences, University of Copenhagen, Frederiksberg, Copenhagen, Denmark; 2 Department of Biology, Faculty of Science, University of Copenhagen, Copenhagen, Denmark; Dalhousie University, Canada

## Abstract

Slick (Slo2.1) and Slack (Slo2.2) channels belong to the family of high-conductance K^+^ channels and have been found widely distributed in the CNS. Both channels are activated by Na^+^ and Cl^−^ and, in addition, Slick channels are regulated by ATP. Therefore, the roles of these channels in regulation of cell excitability as well as ion transport processes, like regulation of cell volume, have been hypothesized. It is the aim of this work to evaluate the sensitivity of Slick and Slack channels to small, fast changes in cell volume and to explore mechanisms, which may explain this type of regulation. For this purpose Slick and Slack channels were co-expressed with aquaporin 1 in *Xenopus laevis* oocytes and cell volume changes of around 5% were induced by exposure to hypotonic or hypertonic media. Whole-cell currents were measured by two electrode voltage clamp. Our results show that Slick channels are dramatically stimulated (196% of control) by cell swelling and inhibited (57% of control) by a decrease in cell volume. In contrast, Slack channels are totally insensitive to similar cell volume changes. The mechanism underlining the strong volume sensitivity of Slick channels needs to be further explored, however we were able to show that it does not depend on an intact actin cytoskeleton, ATP release or vesicle fusion. In conclusion, Slick channels, in contrast to the similar Slack channels, are the only high-conductance K^+^ channels strongly sensitive to small changes in cell volume.

## Introduction

Na^+^ activated potassium currents were first described in 1984 by Kameyama *et al*. [1] in mammalian cardiac cells and five years later they were also found in brain stem neurons by Dryer *et al*. [2]. Thereafter two genes, Slick (**S**equence **L**ike an **I**ntermediate **C**onductance **K** ^+^ channel) and Slack (**S**equence **L**ike **A** **C**a^2+^-activated **K** ^+^ channel), were cloned by Bhattacharjee *et al*. [3] and Joiner *et al*. [4], respectively. These channels have been proposed to have an important role for repolarization of the action potential and for slow afterhyperpolarizations (AHP), which could be of significant importance after episodes of high firing frequency under sustained stimulation [5]. In addition, antiarrhythmic drugs such as clofilium have been found to inhibit Slick and Slack channels, suggesting a potential protective role of these channels in the heart [6]. Both channels are insensitive to Ca^2+^, instead they are activated by Na^+^ and Cl^−^; Slick with higher sensitivity to intracellular Cl^−^ than Slack. Slick channels also possess an ATP binding site in their C-terminus mediating an inhibition of the channels by intracellular ATP [3]. Furthermore, we have recently shown that both channels are modulated by a membrane phospholipid, namely phosphatidylinositol biphosphate (PIP_2_) [7]. Given these particular characteristics, a role for Slick and Slack channels in ion transport processes and regulation of cell volume has been hypothesized [8].

A number of physiological processes, such as salt and water transport, neuronal activity, cell proliferation, migration and apoptosis, result in cell volume changes. Cells often respond to such challenges with a regulatory volume decrease (RVD) or increase (RVI). During a cell swelling induced RVD a loss of osmolytes, such as K^+^ and Cl^−^ and osmotically obliged water, is induced [9]. For some cell types showing a RVD response, an elevation of intracellular Ca^2+^ concentration has been observed during cell swelling [10]. However, there is controversy on the evidence supporting the relationship between the intracellular Ca^2+^ concentration and cell volume changes.

So far, it is not entirely clear which K^+^ channel types should be considered cell volume sensitive and, in particular, the mechanisms for regulation are obscure.

To address the issue of regulation of K^+^ channels by fast and small changes in cell volume, we have co-expressed aquaporins together with a number of K^+^ channel types in *Xenopus laevis* oocytes. The co-expression of aquaporins ensure that the oocytes promptly change their volume, when the extracellular osmolarity is changed. In all cases the results have been very clear. Some K^+^ channels, including KCNQ1, KCNQ4, IK, SK3 and Kir 4.1/5.1, respond with significant increases in current during cell swelling and similar decreases in current during cell shrinkage [11]–[13]. In all cases, the effects have been perfectly reversible, and we consider these channels “sensitive to cell volume changes”. A number of other K^+^ channels, including BK, KCNQ2 and KNCQ3, are in similar experiments totally unaffected by changes in cell volume, and we therefore considered these channels “insensitive to cell volume changes”.

In the present study we provide evidence that Slick K^+^ channels are, so far, the only type of high conductance K^+^ channels, which is strongly regulated by small and fast changes in cell volume, whereas Slack channels, in contrast, are totally unaffected. In addition, we evaluate possible mechanisms, which could explain the tight regulation of Slick channels by cell volume changes. Since Slick channels are insensitive to Ca^2+^, a possible raise in intracellular Ca^2+^ concentration upon cell swelling should not interfere with the hypotonic activation of these channels. We find that the regulation of Slick does not involve interactions with the cytoskeleton, ATP release or vesicle fusion.

## Methods

### Ethics Statement

The procedure to remove oocytes from *Xenopus laevis* frogs was conducted under tricaine anaesthesia (2 g L^−1^) and all efforts were made to minimize animal suffering. This procedure was approved by The Danish National Animal Experiments Inspectorate and was performed in strict accordance with their guidelines.

### Heterologous expression in *Xenopus laevis* oocytes

Preparation of oocytes from *Xenopus laevis* frogs was realized as previously described by Grunnet *et al.* [11]. cDNA encoding hSlick, rSlack into pOX vector (kindly provided by L. Salkoff) and Aquaporin-1 (AQP1) into PBluescript (from P. Agre) were linearized with *Not1* for Slick and Slack or *Pst1* for AQP1 (New England Biolabs, Ipswich, MA, USA). Messenger RNA was obtained by *in vitro* transcription using the mMessage mMachine kit according to manufacturer's instructions (Ambion). RNA was extracted by use of the MEGAclear kit (Ambion). 50 nl. containing approximately 10 ng. of mRNA mixture of Slick with AQP1 or Slack with AQP1 (3∶1 ratio) were injected into oocytes, which were kept in Kulori medium (90 nM NaCl, 1 mM KCl, 1 mM MgCl_2_, 1 mM CaCl_2_, 5 mM HEPES, pH 7.4) at 19°C.

### Electrophysiological measurements

All measurements were performed 4 to 5 days after injection. Currents were measured using a two-electrode voltage clamp amplifier (Warner instruments). Electrodes were pulled from a micropipette puller (P-97, Sutter Instruments CO) and filled with 1 M KCl (resistances were around 0.5 to 1.5 MΩ). Two voltage clamping protocols were used, either a step protocol: −100 to +80 mV in 20 mV steps of 500 ms (holding potential −80 mV for 4 seconds), or by depolarizing pulses of 500 ms from −80 to +80 mV holding the membrane potential at −80 mV for 3 seconds.

Volume measurements were done in isotonic (50 mM D-mannitol, 188 mOsm kg^−1^), hypotonic (0 mM D-mannitol, 137 mOsm kg^−1^) and/or hypertonic media (100 mM D-mannitol, 239 mOsm kg^−1^) all containing 65 mM NaCl, 1 mM KCl, 1 mM MgCl_2_, 1 mM CaCl_2_, 5 mM Hepes, pH 7.4. D-mannitol and Hepes were bought from Sigma and the other chemicals were from Merck.

For evaluating mechanisms for cell volume regulation 10 µM Cytochalasin D or 10 µM Brefeldin A were used to pre-incubate the oocytes for different periods of time up to 24 hs. prior to challenges in cell volume and current measurements. Apyrase (5 U/ml) and ATP (100 µM) were added to isotonic, hypotonic and hypertonic buffers for measuring currents during cell swelling and shrinkage. Cytochalasin D, Brefeldin A, Apyrase and ATP were purchased for Sigma Aldrich.

Data acquisition and analysis were performed using pClamp (Molecular Devices), GraphPad Prism 4 and Excel (Microsoft) packages. Presented data is shown as means ± S.E.M, where statistical differences were evaluated by means of paired Student's *t*-test. *p*-values of less than 0.05 were considered significant.

## Results

### Effect of small changes in cell volume on Slick and Slack K^+^ channels

To analyse the sensitivity of Slick and Slack channels to cell volume changes, both channels were co-expressed in *Xenopus laevis* oocytes together with aquaporin 1 (AQP1), to ensure proper water permeability in the otherwise practically impermeable oocyte membrane. We have earlier shown that the co-expression of AQP1 water channels ensure that the oocytes respond to changes in extracellular osmolarity with fast and significant changes in cell volume [11].

**Figure 1:**
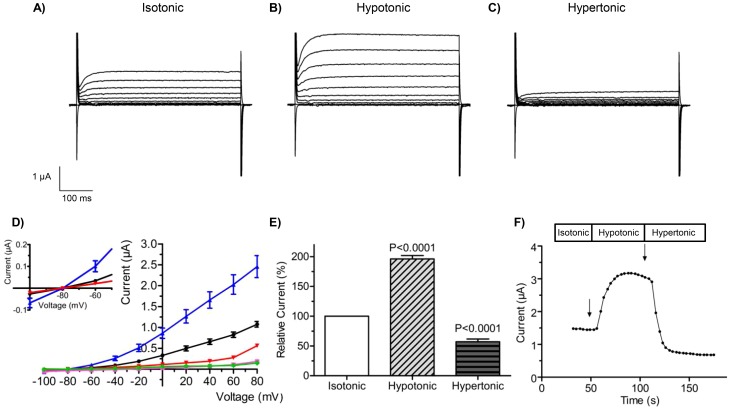
Regulation of Slick channels by small changes in cell volume. In the upper panels, Slick channels were co-expressed with AQP1 in *Xenopus laevis* oocytes and were activated by a step protocol (−100 mV to +80 mV in 20 mV increments of 500 ms) from a holding potential of −80 mV (4 s). Currents were recorded in isotonic (A), hypotonic (B) or hypertonic (C) media. (D) shows the corresponding I/V relationships under isotonic (black), hypotonic (blue) and hypertonic (red) conditions. Control experiments for native, un-injected oocytes (UI, green) and oocytes only expressing AQP1 (violet) are also shown. In (E) maximal currents were measured at the end of the depolarization to +80 mV under isotonic, hypotonic and hypertonic buffers and the currents were normalized to the current measured at isotonic conditions. Data points in panels (D) and (E) show the mean of 10 independent experiments ± S.E.M. (F) displays a current trace over time for a representative oocyte expressing Slick and AQP1. The expressed Slick channels were activated by depolarization to +80 mV (500 ms) from a holding potential of −80 mV (3 s). The Figure shows the current measured at the end of the depolarization period as a function of time. Changes from isotonic medium to hypo- or hypertonic media are marked with arrows in the figure (the apparent delay from change in medium (arrows) to changes in the recorded currents reflect the “dead volume” in the flow system).

Figure 1 (upper panel) shows current traces of an oocyte co-expressing Slick and AQP1 during exposure to isotonic (figure 1A), hypotonic (figure 1B) and hypertonic (figure 1C) media. Slick currents were activated by a step protocol from −100 to +80 mV for 4 seconds with 20 mV increments (holding potential −80 mV). The experiments showed a significant expression of Slick channels; under isotonic conditions (figure 1A) the current measured at +80 mV was 1.07 µA, whereas the current in non-injected oocytes or oocytes expressing AQP1 alone did not exceed 0.15 µA at +80 mV (*data not shown*). After exposure to hypotonic medium (figure 1B) the Slick current was dramatically increased, whereas in hypertonic medium the current was decreased to a similar extent (figure 1C).

Figure 1D shows the I/V relationship for the expressed Slick channels obtained in isotonic medium (black), hypotonic medium (blue) and hypertonic medium (red). I/V curves imply that the voltage dependence as well as the selectivity were not altered during modulation by changes in cell volume. In all cases the currents showed a weak voltage dependence as earlier described [8], and a reversal potential close to the equilibrium potential for K^+^. The I/V relationships for un-injected (UI) oocytes (green) and AQP1 expressing oocytes (purple) indicate that currents originating from endogenous channels in the oocytes did not interfere with our measurements.

The magnitude of the activation of Slick channels during cell swelling, or inhibition during cell shrinkage, were calculated and are shown in figure 1E. A number of oocytes expressing Slick channels together with AQP1 were activated with a pulse protocol from −80 to +80 mV for 3 seconds (holding potential −80 mV) and currents were measured at the end of the depolarizing pulses. During exposure of the oocytes to hypotonic media, K^+^ currents through Slick channels increased to 196% (+/−5%, *n* = 7) compared to control oocytes in isotonic media. In contrast, in hypertonic buffers, Slick currents decreased to 57% (+/− = 4%, *n* = 7) of control. The results of continuous measurements are depicted in figure 1F and show that the responses of Slick channels to either cell volume increase or decrease are instantaneous. As soon as Slick and AQP1 co-expressing oocytes were exposed to hypo- or hypertonic media, activation or inhibition of the currents could be detected. In experiments where oocytes expressing Slick channels without co-expressed AQP1 were exposed to changes in extracellular osmolarity no changes in current could be detected. Previously, we have shown that in the absence of expressed AQP1, oocytes do not show any significant changes in cell volume during exposure to +/−50 mOsm/l changes in osmolarity [12]; these experiments therefore indicate that the activity of the expressed Slick channels is not altered by changes in osmolarity per se (*data not shown*).

**Figure 2:**
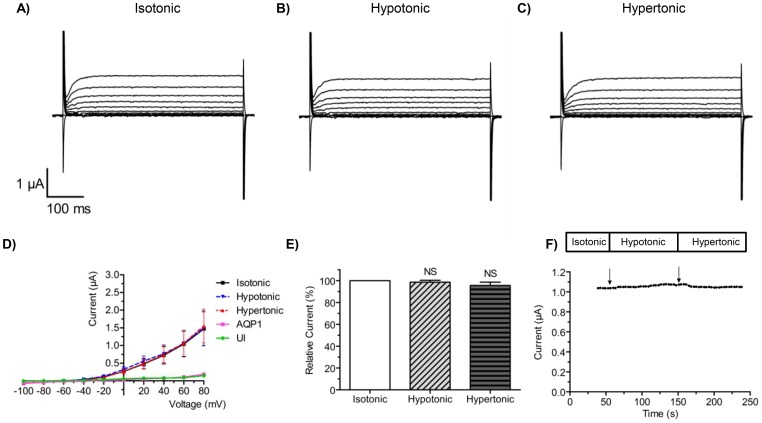
Sensitivity of Slack channels to changes in cell volume. Slack channels were co-expressed with AQP1 in *Xenopus laevis* oocytes and activated with a voltage protocol, as described in figure 1, under isotonic (A), hypotonic (B) and hypertonic media (C). (D) shows the corresponding I/V curves under isotonic (black), hypotonic (blue) and hypertonic (red) conditions. Control experiments for native, un-injected oocytes (UI, green) and oocytes only expressing AQP1 (violet) are also shown. In (E), maximal currents were measured at the end of the +80 mV depolarizing step under isotonic, hypotonic and hypertonic buffers and the currents were normalized to the current measured at isotonic conditions. Data points in panels (D) and (E) show the mean of 10 independent experiments ± S.E.M. (F) shows a current trace over time for a representative oocyte expressing Slack and AQP1. The channels were activated by a protocol as described in figure 1.

In a series of analogous experiments, Slack channels and AQP1 were coexpressed in *Xenopus* oocytes and exposed to hypotonic and hypertonic media (figure 2). Whole-cell currents were unaltered during the induced changes in cell volume (figures 2A, B, C, F) and I/V relationship remained the same after exposure of the oocytes to the different media (figure 2D, n = 8). A comparison of normalized maximal currents in isotonic, hypotonic and hypertonic media also showed no significant differences (figure 2E, n = 9), and finally the continuous measurements (figure 2E) confirmed that the slack current was insensitive to changes in cell volume.

### Kinetics of Slick and AQP1 expressing oocytes during cell volume changes

**Figure 3:**
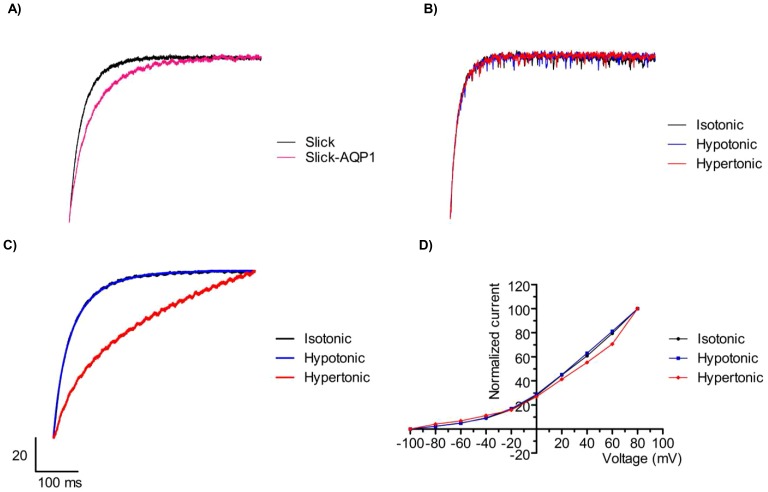
Kinetics of Slick channels during regulation by cell volume changes. (A) Slick channels (Slick, black) or Slick channels and AQP1 (Slick+AQP1, red) were expressed in *Xenopus laevis* oocytes and were kept at a holding potential of −80 mV before the channels were activated by depolarization to +80 mV (500 ms). The recorded currents were normalized to the maximal current measured in each experiment at the end of the +80 mV depolarization period for two representative oocytes. (B) oocytes expressing Slick channels in absence of co-expressed AQP1 were activated by depolarizations to +80 mV as before in isotonic (black), hypotonic (blue) and hypertonic (red) media, and currents were normalized to the maximal current. The figure shows the result of one representative experiment. (C) the experiments shown in (B) were repeated with oocytes co-expressing Slick channels and AQP1 channels (in isotonic (black), hypotonic (blue) and hypertonic (red) media). The figure shows the result of one representative experiment (Note: the currents measured in isotonic and hypotonic media are precisely superimposed). (D) a representative oocyte co-expressing Slick and AQP1 was exposed to isotonic (black), hypotonic (blue) and hypertonic (red) media and in each medium Slick channels were activated by a depolarization protocol as in figure 1. The figure shows the resulting IV-curves, each normalized to the maximal current (found at +80 mV).

Since Slick channels are quite dramatically regulated by changes in cell volume, the following series of experiments were designed to disclose if cell volume changes affect the kinetics of Slick channels. In a first series of experiments, the activation of Slick channels expressed in the presence or absence of AQP1 was compared (figure 3A). Surprisingly, these experiments showed that the activation of Slick was slightly slower in the presence of co-expressed AQP1. These data seem to suggest a possible direct interaction between Slick and AQP1. In a second series of experiments, Slick channels were expressed in *Xenopus* oocytes in the absence of co-expressed AQP1, and the activation kinetics were examined under isotonic, hypo- and hypertonic conditions (figure 3B). The results showed that the channel activation was absolutely unaffected by changes in extracellular osmolarity *per se*. In the experiments shown in figure 3C, it was examined if changes in cell volume affected the activation kinetics of Slick. Therefore, Slick channels were co-expressed with AQP1 and subsequently exposed to the activation protocol in isotonic, hypo- and hypertonic media. Figure 3 C shows the results as normalized currents. In isotonic medium the activation curve (black) already shown in figure 3A (slightly delayed as compared to activation of Slick in the absence of aquaporins) was reproduced, and during cell swelling (hypotonic medium) the activation curve showed no change (blue). However during cell shrinkage (hypertonic medium), there was a significant delay in the activation. Finally the overall I/V relationship for Slick channels during normal cell volume (isotonic medium) and during cell swelling (hypotonic medium) and cell shrinkage (hypertonic medium) was analysed and the results show that the IV-relation is independent on cell volume (figure 3D).

### Mechanisms for cell volume regulation of Slick channels

The mechanism for regulation of K^+^ channels during small and fast changes in cell volume is not yet understood. Therefore, we investigated some hypotheses that could possibly add to our understanding of this phenomenon.

**Figure 4:**
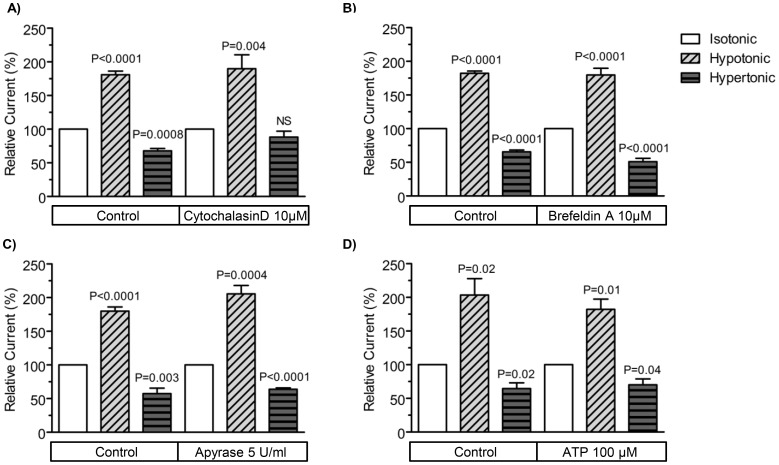
Mechanisms for cell volume regulation of Slick K^+^ channels. Slick channels were co-expressed with AQP1 in *Xenopus laevis* oocytes and the channels were activated by depolarizations to +80 mV for 500 ms (holding potential: −80 mV). In all cases, the maximal currents (at the end of the depolarization period) were measured in isotonic, hypotonic and hypertonic media and are shown as relative changes in the current during cell swelling (hatched bars) and shrinkage (horizontal bars) as compared to the current under isotonic conditions (open bar). The figures show the effect of (A) cytochalasin D (10 µM, preincubation 24 hours), (B) Brefeldin A (10 µM, preincubation 8 hours), (C) Apyrase (5 U/ml) and (D) ATP (100 µM). All values are given as means ±SEM (*n* = 6–8).

We evaluated the role of an intact actin cytoskeleton for the regulation of Slick channels by cell volume. For that purpose, oocytes expressing Slick and AQP1 were pre-incubated with concentrations up to 10 µM of the known F-actin disruptor cytochalasin D, for time periods of up to 24 hours. Treatment of oocytes with this compound did not affect the cell swelling induced activation of Slick channels, but did apparently to some extent reduce inhibition induced by shrinkage (figure 4A).

Next, we investigated if vesicle fusion could account for the strong activation of Slick channels during cell swelling. For these experiments we used Brefeldin A, which is known to block the vesicular transport between the endoplasmic reticulum (ER) and Golgi [14]. In this case we pre-incubated oocytes with 10 µM Brefeldin A for 7 hours before they were exposed to osmotic challenges and currents were measured with a pulse protocol. The results showed that treatment with Brefeldin A did not affect the sensitivity of Slick channels to changes in cell volume (figure 4B).

It has been suggested that ATP release upon cell swelling could also be involved in the volume-induced activation of K^+^ channels through interaction with purinergic receptors [15]. Therefore we tested this hypothesis either by depletion of extracellular ATP with apyrase or by supplementation of media with ATP. The set-up was simple; oocytes expressing Slick channels and AQP1 were exposed to isotonic, hypotonic and hypertonic buffers before and after extracellular addition of the ATP hydrolyzing enzyme, apyrase (5 U/ml) or 100 µM ATP. Neither, depletion of extracellular ATP with apyrase (figure 4C) nor the addition of extracellular ATP (figure 4D) affected the strong regulation of Slick channels by changes in cell volume. In similar experiments, higher concentrations of apyrase and ATP were also tested, showing no effect in the regulation of Slick channels by cell volume (*data not shown*).

## Discussion

### Slick channels are strictly regulated by cell volume, whereas Slack channels are not

In the present study we examined the cell volume sensitivity of two closely related K^+^ channels of the BK channel family, Slick (Slo 2.1) and Slack (Slo 2.2). Both channels were co-expressed with AQP1 in *Xenopus laevis* oocytes to provide the oocyte membrane with the necessary water permeability for immediate swelling and shrinkage when exposed to hypotonic or hypertonic media. We have previously shown that decreasing or increasing osmolarity of the extracellular medium by 50 mOsm results in a cell volume change of approximately 5% within 50 seconds for oocytes expressing AQP1 [11]. The results of the present study show that Slick channels are quite dramatically regulated by such fast, small changes in cell volume; Slick currents were activated to approx. 200% of control during cell swelling and inhibited to approx. 50% of control during cell shrinkage. In contrast, Slack channels are totally insensitive to changes in cell volume. Control experiments showed that in the absence of co-expressed aquaporins, when the oocytes were not able to change cell volume during the described osmotic challenges, the currents through the expressed Slick and Slack channels did not change, indicating that the channels were not sensitive to changes in external osmolarity. As mentioned earlier (cf. Introduction) we have examined the sensitivity of a number of different K^+^ channels to cell volume changes by experiments similar to those employed in the present study. Consequently, we were able to characterize them as sensitive or insensitive to changes in cell volume. The results of the present study clearly show that Slick channels belong to the first group, and that the closely related Slack channels belong to the latter group.

### Possible mechanisms for cell volume regulation of Slick channels

Despite the fact that a number of K^+^ channels have been shown to be precisely regulated by small, fast changes in cell volume, the regulatory mechanism is still obscure. It has been hypothesized that changes in cell volume impose stretch on the cell membrane, and that K^+^ channels apparently sensitive to changes in cell volume, were in fact regulated by changes in membrane stretch. However, we have recently shown that regulation by cell volume and regulation by membrane stretch should be considered two separate regulatory mechanisms: Ca^2+^-activated high-conductance K^+^ channels (BK channels) are strongly activated by membrane stretch, but totally insensitive to cell volume changes, while KCNQ1 channels are strongly sensitive to cell volume changes, but not to membrane stretch [16].

The cytoskeleton is involved at multiple levels in the events that follow cell volume changes. It has been previously shown that disruption of actin cytoskeleton affects channels and transporters during the regulatory volume decrease (RVD) and regulatory volume increase (RVI) processes [17]. In addition, a study of atomic force microscopy, validated by fluorescence microscopy, confirmed the effects of cytochalasin D on the disruption of the actin cortical cytoskeleton in *Xenopus* oocytes treated with this compound [18]. However results are controversial regarding the role of an intact actin cytoskeleton for cell volume regulation. As reviewed by Hoffmann [17], the cytoskeleton is an essential mechanism for the RVD in leukocytes, hepatocytes and Ehrlich ascytes, however it could not explain the cell volume regulation on HL60 cells. Grunnet *et al.* [12] suggested that for KCNQ1 K^+^ channels, an intact actin cytoskeleton is necessary for maintenance of their sensitivity to cell volume. After expression in COS7 cells, however, Piron *et al.* [19] showed that the cell volume sensitivity of the KCNQ1-KCNE channel complex is independent of the actin cytoskeleton. In the present study, we provide evidence that the regulation of Slick channels by cell volume is not coupled to the actin cytoskeleton, since the volume-sensitivity of these channels is not modulated by treatment with cytochalasin.

Osmotic swelling has previously been suggested to involve ATP release in astrocytes, pancreatic and other epithelial cells [20]–[22]. In addition, it has also been shown that *Xenopus laevis* oocytes release ATP during cell swelling [23]. ATP release during cell swelling may activate purinergic receptors (P2Y), which may in turn stimulate ion channels and thus be responsible for the apparent sensitivity to cell volume changes [24]. In the present study we saw no effect in the cell volume sensitivity of Slick channels after the addition of relatively large concentrations of the ATP-hydrolyzing enzyme, apyrase, to the external medium during measurements. Furthermore, addition of extracellular ATP did not affect the response of Slick channels to cell volume changes, and there are no reports that Slick channels should be regulated by purinergic pathways. Although endogeneous purinergic receptors may be expressed in *Xenopus laevis* oocytes, we find it unlikely that a possible ATP release during cell swelling is involved in the sensitivity of Slick channels to cell volume.

Cell swelling or shrinkage has been correlated with stimulation or inhibition of exocytosis, respectively. Changes in vesicle recycling and/or insertion of new proteins in the membrane may account for cell shrinkage induced inhibition and cell swelling induced activation of certain ion channels [25], [26]. Furthermore, physical interaction of SNARE proteins has been shown to activate K_ATP_ and K_V2.1_ channels [27]. Brefeldin A is a compound known to suppress vesicular transport between the endoplamastic reticulum (ER) and Golgi [14], thereby blocking transport of newly synthesized proteins from the ER and preventing translocation to the plasma membrane. The present study shows that after incubation of *Xenopus* oocytes with Brefeldin A, blockage of the exocytic pathway does not affect the volume response of Slick channels and it seems not likely that cell volume changes modulate the number of these channels in the membrane. These results are consistent with our findings regarding the role of the cytoskeleton, since an intact actin cytoskeleton would be needed for proper transport of vesicles to the cell membrane. Thus, we find it a more attractive hypothesis that changes in cell volume regulate Slick channels, which are already present in the cell membrane.

Most interestingly, it has recently been suggested by Piron *et al.* [19] that the strong cell volume regulation of KCNQ1/KCNE1 channels is mediated through interaction with PIP_2_ (phosphatidylinositol 4,5-biphosphate). In addition, these authors suggest that PIP_2_-interaction, in general, may play an important role for regulation cell volume sensitive K^+^ channels. Indeed, a number of cell volume sensitive K^+^ channels, including KCNQ3, KCNQ4 and K_ir_ channels have also been shown to be regulated by PIP_2_ [28], [29]. According to the hypothesis of Piron [19], the intracellular concentrations of Mg^2+^ and polyamines are diluted during cell swelling resulting in a reduced interaction of these cations with PIP_2_, which in turn leads to an enhanced stimulatory interaction of PIP_2_ with the apparently volume sensitive K^+^ channels. However, if this would indeed be the mechanism, we could expect that KCNQ2/3, which are also sensitive to PIP_2_, Mg^2+^ and polyamines, would also be sensitive to changes in cell volume, but these channels have been proven to be totally insensitive to cell volume [12]. Given the results of the present study, this hypothesis would require PIP_2_ sensitivity of Slick channels, but not of Slack channels. However, our previous findings indicate that both channels are equally activated by PIP_2_, suggesting that the volume sensitivity of Slick channels is not based on a PIP_2_ related mechanism [7].

### Interaction between K^+^ channels and aquaporins

The fact that certain K^+^ channels, when co-expressed with aquaporins in *Xenopus* oocytes respond to changes in extracellular osmolarity and the resulting small, fast changes in cell volume with large changes in current, can be described as a *functional* interaction between ion channels and aquaporins. We have until now expected that the role of the aquaporins is merely to ensure a high water permeability, which will allow the fast changes in cell volume to take place. In contrast, if aquaporins are not co-expressed, the channels are totally insensitive to changes in extracellular osmolarity, simply because the cells do not change their volume significantly. So far, we have not had any evidence for a *direct* interaction between the channels and aquaporins. However, if figure 3A is inspected, the measurements show that the activation kinetics are surprisingly slightly slowed by the co-expressed aquaporins. Based on this minimal change in channel kinetics induced by the presence of aquaporins, we find it tempting to suggest a direct interaction between Slick channels and APQ1. In fact, at present we cannot think of any other explanation for this phenomenon. However this intriguing suggestion should be carefully examined in future experiments.

### Physiological relevance of the regulation of Slick channels by cell volume changes

Slick channels have been identified in CNS, where they may associate either with BK or Slack to form heteromeric channels [4], [30]. Slick channels seem to play an important role in modulation of the neuronal afterhyperpolazation [31] and expression of these channels has also been found in cardiac cells, skeletal muscle, kidney, lung, liver, testis and pancreas [32], [33]. The possible protective role of activated Slick channels during episodes of hypoxia/ischemia has been examined by Yuan *et al.* [33]. In the present study we have found that Slick channels are strongly activated by a small increase in cell volume and are inactivated by a similar cell-volume decrease. If Slick channels should be ascribed a protective role during ischemia, our results suggest that in such cases, the channels could not only be activated by increased Na^+^ and decreased ATP [3], but also by the cell swelling, which is, inevitably, a consequence of ischemia.

## Conclusions

In conclusion our work reveals that Slick and Slack K^+^ channels, despite their high homology are differently regulated by cell volume changes. Slick channels are strongly activated upon a small increase in cell volume and inhibited by a similar decrease in cell volume. Slack channels, on the other hand, are totally insensitive to cell swelling or shrinkage. We have provided evidence that the cell volume sensitivity of Slick channels does not depend on an intact actin cytoskeleton, ATP release or vesicle fusion. However, the mechanism responsible for the volume sensitivity of Slick channels and of other K^+^ channels is still not clear and should be further explored. In this context, it is noteworthy that functional chimeras may be constructed between Slick and Slack channels and we anticipate that such chimeric channels may, in future studies, act as an important tool for exploration of the structure-function relationships of cell volume sensitive K^+^ channels. The physiological relevance of our findings regarding the regulation of Slick channels by small and fast changes in cell volume, considering the localization of these channels in e.g. the CNS and possibly in the heart, is the possible role of these channels during the pathological cell swelling that accompanies episodes of ischemia.

## Supporting Information

Dataset S1 **Datasets available at the Repository of the University of Copenhagen, CURIS.**
(DOCX)
