# Supplementary material for: Cell Volume Changes Regulate Slick (Slo2.1), but Not Slack (Slo2.2) K+ Channels
Source: PLoS One. 2014 Oct 27;9(10):e110833. doi: 10.1371/journal.pone.0110833 (PMC4210196; doi:10.1371/journal.pone.0110833)
Supplement: Dataset S1 — Datasets available at the Repository of the University of Copenhagen, CURIS. (DOCX) [file pone.0110833.s001.docx]

DATASET S1

Datasets available at the Repository of the University of Copenhagen. (CURIS)

Tejada et al. Cell volume changes regulate Slick (Slo2.1), but not Slack (Slo2.2) K+ channels

<http://curis.ku.dk/portal/files/123607354/Fig1VolRegSlickSlack_MT2014.zip>

<http://curis.ku.dk/portal/files/123607355/Fig2VolRegSlickSlack_MT2014.zip>

<http://curis.ku.dk/portal/files/123607356/Fig3VolRegSlickSlack_MT2014.zip>

<http://curis.ku.dk/portal/files/123607357/Fig4VolRegSlickSlack_MT2014.zip>
